# Validation of OSLER Test Against Maintenance of Wakefulness Test and the Association With Subjective Measures of Sleepiness

**DOI:** 10.1111/jsr.70251

**Published:** 2025-11-22

**Authors:** Riikka Huhta, Anniina Alakuijala, Markku Partinen

**Affiliations:** ^1^ Department of Clinical Neurosciences University of Helsinki Clinicum Unit Helsinki Finland; ^2^ Helsinki Sleep Clinic Terveystalo Healthcare Services Helsinki Finland; ^3^ Department of Clinical Neurophysiology, HUS Diagnostic Center Helsinki University Hospital and University of Helsinki Helsinki Finland

**Keywords:** alertness, MWT, OSLER test, professional drivers, sleepiness, vigilance (max 6kpl)

## Abstract

The ability to stay awake is crucial in life and can be compromised by insufficient sleep and medical conditions. Measuring alertness is important for evaluating driving ability and the Oxford Sleep Resistance (OSLER) test may provide an easy way for assessment. The study population included 40 professional truck drivers who participated in the Maintenance of Wakefulness Test (MWT) between 2000 and 2002. Half of these subjects had sleep latency less than 33 min in the MWT. All subjects completed a questionnaire, underwent interviews, participated in both the MWT and OSLER test on consecutive days and filled in the Karolinska Sleepiness Scale (KSS) before each MWT and OSLER test session. Altogether, 39 subjects successfully completed both the MWT and OSLER test in random order. The MWT took place after polysomnography. The mean sleep latency in the MWT was 38.7 min while the mean sleep latency in the OSLER test was 38.9 min. If the sleep latency in the OSLER test exceeded 33 min, it was highly likely that the sleep latency in the MWT would also be normal (> 33 min). The OSLER test appears to be a reliable objective measure for assessing the ability to stay awake in professional drivers and is easier to administer than the MWT, which still remains the gold standard.

## 1 Introduction

Daytime alertness is important for managing everyday life. Working and driving abilities depend on it. There are plenty of situations that make it difficult to stay awake during the day. The most important of these reasons is sleep deprivation, but conditions such as narcolepsy or sleep apnea can also cause daytime sleepiness (Liu et al. 2000; Lowe et al. 2017; Gandhi et al. 2021). Objective tools to measure the ability to stay awake during the day are important.

The Maintenance of Wakefulness Test (MWT) is a test to measure the ability to stay awake (Doghramji et al. 1997; Moldofsky 1992). It is a gold standard for measuring alertness and ability to drive, although it is still unsure how it really predicts driving ability (Sagaspe et al. 2007; Virtanen et al. 2019).

The Oxford SLEep Resistance Test (OSLER Test), a behavioural test, is designed to evaluate the ability to stay awake in the same way that the MWT does (Bennett et al. 1997). It does not require EEG measurement and is therefore less expensive and easier to accomplish. A couple of studies with small populations have evaluated it compared to the MWT, and the results look promising (Krieger et al. 2004; Priest et al. 2001). It has also been studied whether the measurement of the number of missed responses and error profiles in the OSLER test could add to the reliability of the test result compared to measuring only the sleep latency (SL) (Mazza et al. 2002). OSLER results have been shown to reveal responses to continuous positive airway pressure (CPAP) treatment in patients with obstructive sleep apnea (OSA) (Alakuijala et al. 2014). One group has introduced a software‐based Behavioural Sleep Resistance Task, which is more or less the same test with different equipment and seems to work as well as the original OSLER test (Mairesse et al. 2009). In 2021, one group published data from their study using the same protocol with the flashing light inside dark glasses and compared it with the OSLER Test (Larrateguy et al. 2021). In a modified OSLER test, patients with OSA with more missed stimuli have more motor vehicle accidents (Karimi et al. 2015).

According to European Union directives, the ability to stay awake needs to be evaluated among drivers with a group 2 driver's licence who have at least moderate sleep apnea with an apnea‐hypopnea index (AHI) of 15 or higher (Commission directive 2014/85/EU) (Bonsignore et al. 2016). The occurrence of sleep apnea in professional drivers is much greater than in the general population (Huhta et al. 2021). Hence, there is a need for practical objective tests of the ability to stay awake.

The OSLER test has been used by clinicians for testing daytime alertness and to evaluate one's ability to drive. Despite that, the data on the usefulness of the OSLER test rely on a couple of small populations. In this study, our aim was to validate the OSLER test in professional drivers. The subjects attended both the MWT and the OSLER test on consecutive days. We also aimed to evaluate the association between objective and subjective tests of alertness.

## 2 Subjects and Methods

### 2.1 Subjects

The population of this study consisted of professional truck drivers who successfully participated in the MWT during the Unikuorma‐study in 2000–2002. Back then, they lived in Southern Finland and were picked from the registry of Finnish Truck Drivers (Rahtarit Ry). We excluded those who were no longer living in Nordic countries and collected data on deceased individuals from the Population Information System. We included only men because only a few women participated in the original study.

We divided the subjects into two groups according to their previous MWT result as follows: Group A: those with SL in MWT < 33 min and Group B: those with MWT ≥ 33 min. We chose randomly 20 subjects from each group. We chose this grouping to improve the possibility of getting both alert and less alert subjects for this study.

### 2.2 Contacting the Subjects

All professional truck drivers examined in the Unikuorma study with MWT were contacted by phone approximately 14 years later. The questionnaire used in the interviews was modified from the BNSQ (Basic Nordic Sleep Questionnaire) and included questions about weight, height, illnesses, sleep apnea, other sleep disorders, snoring, witnessed apneas, medication, daytime sleepiness, and it also included the Epworth Sleepiness Scale (ESS). A validated depression questionnaire (DEPS) was used to screen for depressive symptoms (Salokangas et al. 1995). Additionally, habits like smoking or alcohol consumption, kilometres driven per year, and traffic accidents were asked about. We also asked subjects to fill in the Karolinska Sleepiness Scale (KSS) before each MWT and OSLER test session.

### 2.3 Maintenance of Wakefulness Test

MWT was performed after a full‐night polysomnography according to AASM criteria and using the limits of Doghramji, Philip, and Sagaspe (Doghramji et al. 1997; Philip et al. 2021; Sagaspe et al. 2007). In MWT, the subject is seated in a darkened room (5 lx), in a semi‐recumbent position, and asked to stay awake without using any specific tools for that. The 40‐min session takes place four times every 2 h. In our study, the first session started between 8 and 9 a.m., and if the subject had not yet woken up, the nurse woke him up no later than 1.5 h before the first MWT session. After the second session, subjects ate a standardized lunch.

EEG, electrooculography (EOG), submental electromyogram (EMG), and eye movements were registered. Face and eyes were recorded by the camera integrated into the Somnoscreen system (Somnomedics, Germany). If the subject fell asleep, he was awakened after 90 s of stage 1 sleep or after one 30‐s epoch of any other stage of sleep. SL of each MWT session was defined as the time from the beginning of the session to the beginning of the first epoch of sleep. The test result was defined as an average of the sleep latencies of all four sessions. If the subject did not fall asleep, the test was terminated after 40 min, and then the sleep latency of this session was 40 min.

### 2.4 Oxford Sleep Resistance Test

As mentioned above, the subjects also underwent the OSLER test. It was performed either the day before or the day after the MWT. The order was randomly selected, and there were 50% of the subjects in each group.

In the OSLER test, the circumstances are very similar to those of the MWT. The sitting position, lighting, and the settings of the four 40‐min sessions are the same. An OSLER test does not include the registration of EEG, EOG or EMG, but the face and eyes are recorded by the camera in the same way. Instead, the LED light is placed at the same height but 2.5 m away from the subject's eyes. The LED light flashes every 3 s for a second, and the subject is asked to press the button placed in his hand every time the light appears. The test is terminated after seven consecutive missed lights or after 40 min. The average of the results of all four sessions is used as a test result. In our study, the first session of the OSLER test started between 8 and 9 a.m., similar to that in MWT. A standardized lunch was served after the second session. We also calculated the number of missed stimuli as an OSLER error index (Mazza et al. 2002).

### 2.5 Statistics

The statistical analysis of the data was carried out with Stata 17.0 for Mac (StataCorp, College Station, TX, USA). Before starting the study, we conducted power computations using nQuery to estimate the sample size. We ended up including 40 subjects, which allowed 90% power with *p* < 0.05. We provide means, standard deviations, medians, and ranges. The normality of distributions was tested by the Shapiro–Wilk tests. Parametric Student *t*‐tests and non‐parametric Mann–Whitney *U*‐tests were used according to distributions. Pearson's pairwise correlations were done using the Bonferroni correction. Spearman correlations were also computed. In the comparison of the results between different MWT and OSLER sessions, we used post‐estimation contrasts. Only subjects with complete information were included in the analyses.

## 3 Results

Forty subjects attended this OSLER test validation study. One of the subjects performed poorly in the OSLER test because he had trouble understanding the instructions. His test had to be terminated because of this, and his results have been excluded from the analysis. The mean age of the remaining 39 subjects was 53.7 years (range 35–71 years, SD 10.0 years, median 52.3 years). The mean body mass index (BMI) using the measured weight and height was 30.6 kg/m^2^ (range 23.0–46.2 kg/m^2^, SD 6.1 kg/m^2^, median 28.7 kg/m^2^).

Forty‐four percent of the subjects reported having hypertension and 19% of the subjects had diabetes. One subject had coronary artery disease. Fifteen drivers (38%) knew they suffered from sleep apnea before this study, and three of them (21%) had been using CPAP before the study. In the polysomnography (PSG), the mean AHI was 21.3 (SD 20.6, range 1.6–82.7). AHI was ≥ 5 in 77% and ≥ 15 in 46% of the subjects, respectively.

Of the subjects, 79.5% reported snoring at least sometimes, 46.2% were snoring at least three nights a week, 35.1% reported they had breathing pauses at least sometimes, and 10.8% reported having apneas almost every night or every night. Sixteen percent of the subjects reported having restless legs syndrome. None of the subjects had clinically relevant symptoms of depression according to the DEPS test.

### 3.1 Subjective Sleepiness

The Epworth sleepiness scale (ESS) was used to measure subjective sleepiness. The mean ESS score was 6.4 (range 1–15, SD 3.2, median 6). Only 7.7% of the subjects had an ESS score ≥ 11, which is considered abnormal.

In response to the question, “Do you feel sleepy during daytime?” 33.3% answered they felt sleepy at least once a week. 15.4% answered they felt sleepy at least three days a week.

Subjects filled in the KSS before every MWT and every OSLER test session. In OSLER test session one, 76.9% of the subjects reported being at least rather alert (KSS < 5), and in the other sessions, the percentages were 89.7% in session two, 79.5% in session three, and 82.1% in session four. Nobody reported needing any effort to stay alert before any OSLER test session.

Respectively, before MWT session one, 74.4% of the subjects reported being at least rather alert; before session two, the percentage was 89.7%; before session three, 84.6%; and before session four, 87.2%.

### 3.2 MWT

All 39 subjects participated in MWT after the full‐night PSG. 71.8% of the subjects had the maximum result in MWT, which means 40 min. Only 7.7% had a result < 33 min. The mean SL in MWT was 38.7 min (range 23.9–40 min, SD 3.2 min, median 40 min). All drivers had an SL ≥ 19.4 min.

All three subjects using CPAP treatment before this study had MWT results ≥ 33 min. Two of them had the maximum MWT result (40 min), and one had 38.4 min.

Of those with AHI ≥ 15 two had MWT result < 33 min and also OSLER < 33 min. One of those with AHI < 15 had MWT result < 33 min, but none had OSLER < 33 min. Of those with AHI ≥ 5 two had MWT results < 33 min and two had OSLER < 33 min. One of those with AHI < 5 had MWT < 33 min. Nobody with AHI < 5 had OSLER < 33 min. The differences between MWT and OSLER results were not statistically significant.

### 3.3 OSLER Test Validation

Altogether, 40 subjects participated in the OSLER test. The OSLER test result of one subject who performed poorly (because of cognitive difficulties) was only 7.7 min, whereas his MWT result was 40 min. This SL in the OSLER test was much lower than that of the other participants. Including his result, the mean OSLER test SL was 38.1 min (range 7.7–40 min, SD 5.9 min, median 40 min), and if excluded, the mean OSLER test SL was 38.9 min (range 24.4–40 min, SD 3.3 min, median 40 min).

As with the MWT, all three subjects using CPAP treatment before this study had OSLER test results ≥ 33 min. In fact, two of them had OSLER test results of 40 min, and one had 39 min.

Table 1 depicts the main MWT and OSLER results and their correlations. Only 5.1% of the subjects had OSLER test SL < 33 min. With this amount of abnormal test results, the sensitivity of the OSLER test against MWT was 50.0%, and the specificity was 94.6%. If the number of abnormal OSLER test results had been greater, the sensitivity and specificity would have been better (with 17.3% prevalence of OSLER test results < 33 min, the sensitivity would have been 98.7% and the specificity 99.3%). We also analyzed the correlation between OSLER test and MWT with the result < 38 min, and the results were similar.

**TABLE 1:** MWT and OSLER results.

	MWT	OSLER	*r*	*p*
*n*	39	39		
SL mean (SD) (min)	38.7 (3.2)	38.9 (3.3)	0.316^a^	< 0.001^b^
SL range (min)	23.9–40.0	24.4–40.0		
SL < 38 min (%)	18.0	10.3		0.563^c^
SL < 33 min (%)	7.7	5.1		0.150^c^

Abbreviations: *r*, correlation coefficient; SL, sleep latency.

^a^ Spearman's rho.

^b^ Wilcoxon signed‐rank test.

^c^ Fisher's exact test.

76.9% of the subjects had the maximum result, 40 min. Only five subjects had test results < 40 min in both tests, OSLER and MWT. 94.6% of the subjects had results ≥ 33 min in both MWT and OSLER tests.

**FIGURE 1:**
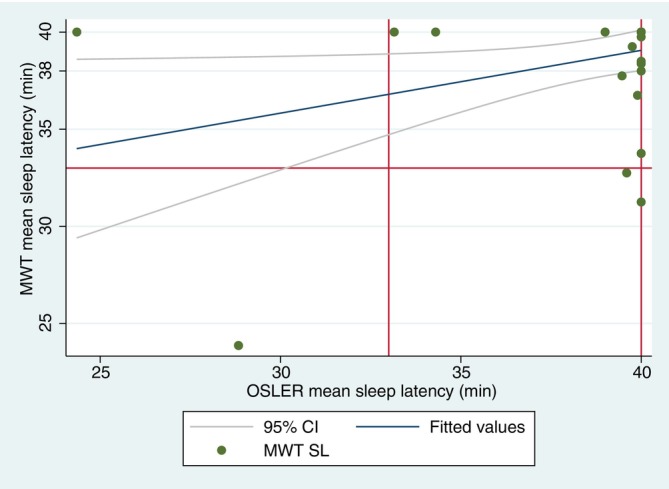
MWT against OSLER sleep latencies.

When excluding the subject with cognitive difficulties mentioned above, the MWT result correlated weakly with the OSLER test result (MWT = 25.00 + 0.353*OSLER result; standard error 0.150). As demonstrated in Figure 1, if the OSLER test SL is > 33 min, the MWT result is also likely to be normal (> 33 min). In contrast, the OSLER test SL < 33 min did not correlate well with the MWT SL. In a pairwise Spearman correlation test, the MWT sleep latency and OSLER sleep latency were independent (*r* = 0.316; *p* = 0.064).

We did computations of error profiles in the OSLER test as well. On average, the subjects made 1.5 errors during one OSLER test session (SD 0.2). The mean error amount in session one was 1.1 (SD 0.3), in session two 1.4 (SD 0.3), in session three 1.5 (SD 0.3), and in session four it was 1.8 (SD 0.4). Error amount tä means the total number of errors in all four sessions divided by four (the number of sessions). The correlation between error amount and OSLER test result was statistically significant (*p* = 0.0001), but it was not statistically significant between error amount and MWT result (*p* = 0.2493).

Those subjects with SL in the MWT ≥ 33 min had a mean error amount of 1.4, and those with MWT SL < 33 min had 2.8 (SD 1.4). Those subjects with OSLER test SL < 40 min had a mean error amount of 3.4 (SD 0.5), and those with OSLER test SL ≥ 40 min had 0.9 (SD 0.2). Those subjects with OSLER test SL < 33 min had a mean error amount of 4.0 (SD 1.3), and those with OSLER test SL ≥ 33 min had 1.3 (SD 0.2).

We also calculated the OSLER error index (error amount per hour), which was on average 2.69 (SD 3.3). Those subjects with MWT SL ≥ 33 min had a mean OSLER error index of 2.4 (SD 2.7), and those with MWT SL < 33 min had 6.4 (SD 7.3). Those subjects with OSLER test SL < 40 min had a mean OSLER error index of 6.9 (SD 4.3), and those with OSLER test SL = 40 min had 1.4 (SD 1.3). Those subjects with OSLER test SL ≥ 33 min had a mean OSLER error index of 2.2 (SD 2.5), and those with OSLER test SL < 33 min had 11.1 (SD 5.1).

Next, we checked whether the order of the two tests, OSLER test and MWT, affected the test results. If OSLER was done before MWT, the mean SL of OSLER was 39.08 min (SD 2.7) with a mean OSLER error index per hour of 2.8 (SD 3.6). If OSLER was done after MWT, the mean SL of OSLER was 38.78 min (SD 3.8) with a mean OSLER error index of 2.6 (SD 3.0). The respective values for MWT were 38.69 min (SD 3.8) and 38.75 min (SD 2.5). That difference was not statistically significant. For OSLER test results, *p* = 0.819 (Mann–Whitney *U*‐test). For MWT results, *p* = 0.373 (Mann–Whitney *U*‐test). We also checked if the total sleep time on the night preceding MWT had an effect on the OSLER test or MWT results, but it did not.

The subjects had the shortest sleep latency in the third session of the OSLER test (right after lunchtime), and the same observation was true with the MWT sleep latency. However, this difference was not statistically significant.

As a whole, the best results were found in the 2nd test, and the worst average results were found in the 3rd session, as shown in Table 2. Although there were no statistically significant differences between different groups, there were statistically significant differences in the pairwise intraindividual comparisons in the MWT results. Using contrasts, the MWT results of the 3rd session were statistically significantly worse than the MWT results in the 1st or 2nd session. The MWT results of the 3rd and 4th sessions did not differ statistically significantly. The differences in the OSLER results did not differ statistically significantly between different sessions, although the worst results were found in the 3rd session.

**TABLE 2:** Pairwise comparisons of the MWT sessions.

Test session #	MWT (min) median; mean (SD); range	Osler median; mean (SD); range
1st	40; 39.2 (3.2); 21.5–40	40; 38.7 (4.5); 17.4–40
2nd	40; 39.5 (2.9); 22–40	40; 40.0 (0.2); 38.9–40
3rd	40; 36.5 (8.6); 0–40	40; 38.8 (5.0); 14.5–40
4th	40; 38.5 (6.4); 8.5–40	40; 39.0 (3.6); 22–40

When considering the subjective measures of sleepiness, we tested the correlation of OSLER test and MWT with ESS and KSS. The mean ESS of those with SL in the MWT < 33 min was 11 (SD 3.1), and of those with SL in the MWT ≥ 33 min it was 6.0 (SD 0.5). The mean ESS of those with OSLER test SL < 33 min was 8.5 (SD 4.5), and of those with OSLER test SL ≥ 33 min it was 6.2 (SD 0.5). ESS did not correlate with either OSLER test result (*p* = 1.00, Pearson *r* = −0.066, Spearman *r* = −0.182) or the MWT result (*p* = 0.0535, Pearson *r* = −0.413, Spearman *r* = −0.325). There was also no significant correlation between KSS and either MWT result or OSLER test result (data not shown). Of those who had OSLER test SL < 33 min, no one reported being alert, very alert or extremely alert before any session (which means that they scored at least four points in KSS), but before some of the sessions reported being rather alert (KSS = 4). Among those who had MWT result < 33 min, there was more variability in KSS.

## 4 Discussion

The OSLER test is suitable for assessing the ability to stay awake in professional drivers, at least if the test result is normal. In this study, the professional truck drivers performed very well in both the OSLER test and MWT. The mean OSLER test sleep latency was over 38 min, as it was also in MWT. Their OSLER error index was, on average, remarkably better than that of clinical sleep unit patients in an earlier study (Mairesse et al. 2009). If we use a cut‐off point of 33 min as the limit of normal ability to stay awake, the MWT result associated well with the OSLER test result, even though the correlations of the absolute sleep latencies were poor.

If the OSLER test result is normal (SL > 33 min), the probability of a normal alertness level is high. On the contrary, if the OSLER test result is abnormal (SL < 33 min), there is a possibility of a falsely abnormal result. In such a case, one could possibly check from the camera showing the face and eyes, whether the subject has really fallen asleep or not. If circumstances are unclear, one should not make inferences about the professional driver's tendency to fall asleep based solely on the OSLER results. Instead, in real life, the driving history of the driver must be considered (Sagaspe et al. 2010). If there have been near‐miss events or some other problems related to sleepiness at the wheel in the driver's history, the OSLER test or MWT should be repeated to ascertain that there are no significant problems in the ability to stay awake. On the other hand, in some cases, the OSLER test result may be more realistic than the MWT result. According to scoring rules in the MWT, if the subject falls asleep at the end of one epoch and wakes up at the beginning of the next one, both epochs can be scored as wake and the test session as normal, even if the subject was actually asleep for 25 s. The same time lapse spent asleep in the OSLER test would cause seven missed stimuli and a truthful abnormal result.

It seems that if we only use one session of either one of the tests, the third session right after lunch would have the shortest sleep latency, and it might be a good idea to use this session only. However, this observation needs more investigation.

One of the reasons for worse results in the OSLER test than in the MWT in our study was cognitive problems that made it harder for the subject to understand the instructions. One reason was neuronal damage in the index finger preventing proper use of this finger. One can conclude that OSLER is not a suitable option for measuring vigilance among subjects with cognitive or fine motor problems. One of the reasons for poor OSLER results might also be a lack of motivation. It would be easy to simply stop pressing the button and thus get a poor result (Alakuijala et al. 2021). Bonnet et al. have also studied the effect of motivation in MSLT and MWT and found that motivation can decrease the sleep latency in the MWT as well (Bonnet and Arand 2005). We did not seem to have this motivation problem in our study.

In previous studies, the OSLER test has been shown to be an appropriate test in evaluating daytime alertness. In the original study in 1997, Bennett et al. (1997) studied 10 subjects suffering from severe symptomatic sleep apnea and compared their MWT and OSLER test results with 10 normal controls and found this method to separate these groups from one another as well as the MWT did. Krieger et al. (2004) studied 11 participants with the MWT and the OSLER test in 2004 and found a good correlation of MWT and OSLER test sleep latencies. Huang et al. (2020) compared the OSLER test result with the MSLT result in 2020 and found no correlation between these two tests. They also evaluated the same subjective tests as we did, ESS and KSS, and found no correlation between these two tests and MSLT or OSLER test.

Priest et al. studied in 2001 whether the number of missed stimuli in the OSLER test is remarkable, and they found that microsleep was present almost always after four consecutive missed stimuli (Priest et al. 2001). They also suggested that the number of missed stimuli in the OSLER test could be an extra valuable marker of reduced alertness. They also found that the subject could even continue to push the button during the microsleep. Alakuijala et al. (2021) studied the factors associated with abnormal OSLER test results and found that the subjective estimate of one's work capability correlated with the result the best.

All our subjects were professional truck drivers, allowing us to make inferences on the use of the MWT and OSLER test in that population. Our population represents well truck drivers in Finland, and we have no reason to think that the Finnish truck driver population differs from truck drivers in other countries in terms of age, gender, and body composition (e.g., presence of obesity). We had 39 subjects, which is more than in most previous studies (Bennett et al. 1997; Krieger et al. 2004; Priest et al. 2001). The subjects were selected randomly, and the order of the tests (MWT and the OSLER test) was random. One of the limitations is that because all our subjects were professional drivers, the results cannot be generalized directly to the general public. We had only a few subjects with abnormal test results altogether since most of the subjects had the maximum result in both the OSLER test and the MWT. This might mean that professional drivers are able to stay alert in monotonous situations better than other populations. This would be easy to understand in the context of their professional demands. Whether professional drivers really are more alert at the wheel than non‐professional drivers, was addressed in the study of Anund et al. (2018). At least professional drivers felt themselves less sleepy while driving, but the objective evaluation remains unclear (Anund et al. 2018). The other question is, whether the MWT is a good method to predict driving ability, which was studied by Virtanen et al. (2019). This needs to be studied further, but at the moment the MWT is the test of choice also recommended by the EU commission (Bonsignore et al. 2016). Also, we only had men in our study population. However, professional drivers are much more often men than women in real life, too.

This study shows that the OSLER test can be used to assess the ability to stay awake, and in that way, it can be used to assess the driving ability of professional drivers with obstructive sleep apnea. One problem is that the original OSLER test by Stowood Inc. is no longer commercially available. Therefore, some mimics have been developed, for example, in Finland. The MWT can still be considered the test of choice to estimate the ability to stay awake among professional drivers. Hopefully, simpler tools, such as the OSLER test, will be commercialized and adequately validated by some company. Compared to the MWT, the OSLER test could save time for the healthcare professional in analysis, and money with less technical requirements for the device and less personnel costs because no certified polysomnographic sleep technologists are needed to monitor the test.

In future studies, it is important to validate the OSLER test in a larger population of drivers other than professional drivers only and among more sleepy subjects than studied for the present. Also, testing whether only one to three test sessions would be enough is worth evaluating further. This is of great value, especially when considering the economic aspects of healthcare.

## Author Contributions

**Riikka Huhta:** conceptualization, data curation, formal analysis, funding acquisition, investigation, methodology, project administration, validation, visualization, writing – original draft preparation, writing – review and editing. **Anniina Alakuijala:** writing – review and editing. **Markku Partinen:** conceptualization, data curation, formal analysis, funding acquisition, investigation, methodology, project administration, resources, supervision, validation, writing – review and editing.

## Funding

This work was supported by the Finnish Work Environment Fund, 114074; Henry Ford Foundation; Maire Taponen Foundation; the Finnish Sleep Research Society; the Research Foundation of the Pulmonary Diseases; the Finnish Traffic Medicine Foundation and Väinö and Laina Kivi Foundation.

## Ethics Statement

This study was approved by the ethical committee of Helsinki University Hospital.

## Consent

Informed consent was obtained from all subjects involved in the study.

## Conflicts of Interest

Riikka Huhta reports grants and personal fees from the Finnish Work Environment Fund, grants from Henry Ford Foundation, grants from the Maire Taponen Foundation, grants from the Finnish Sleep Research Society, grants from HES, grants from the Research Foundation of the Pulmonary Diseases, grants from the Finnish Traffic Medicine Foundation, and grants from Väinö ja Laina Kivi Foundation, during this study. Anniina Alakuijala declares no conflicts of interest. Markku Partinen reports grants from Signe and Arne Gyllenberg Foundation, other from Takeda, other from Orion, other from Teva, other from Lundbeck, outside the submitted work.

## Data Availability

The data that support the findings of this study are available from the corresponding author upon reasonable request.
